# ‘I don’t think they really link together, do they?’ An ethnography of multi-professional involvement in advance care planning in nursing homes

**DOI:** 10.1093/ageing/afad234

**Published:** 2023-12-28

**Authors:** Nicola Andrews, Michelle Myall

**Affiliations:** School of Health Sciences, University of Southampton, Southampton SO17 1BJ, UK; School of Health Sciences, University of Southampton, Southampton SO17 1BJ, UK

**Keywords:** advance care planning, multi-disciplinary team, nursing homes, family, ethnography, qualitative research, older people

## Abstract

**Background:**

Given the globally ageing population, care homes have an important role in delivering palliative and end-of-life care. Advance care planning (ACP) is promoted to improve the quality of end-of-life care in this setting. While many professionals can be involved in ACP, little is known about what influences multi-professional involvement and how multi-professional working impacts the ACP process in the UK. This study investigated multi-professional practice in relation to ACP in nursing homes.

**Design and methods:**

An ethnography was undertaken in two UK nursing homes using multiple methods of data collection: observations, interviews and document review. Participants included the following: nursing home residents (*n* = 6), relatives (*n* = 4), nursing home staff (*n* = 19), and visiting health and social care professionals (*n* = 7). Analysis integrated thematic analysis, mapping of resident ACP trajectories and documentary analysis.

**Findings:**

This paper suggests that multi-professional and relatives’ involvement in ACP was disjointed. Continuity and coordination were disrupted by misalignment of visiting professional and nursing home organisational structures. Findings show a ‘knotworking’ approach to teamwork and power imbalance between nursing home staff and visiting professionals, such as general practitioners. While residents wished their relatives to be involved in their ACP, this was not formally recognised, and limited support existed to facilitate their involvement.

**Conclusion:**

The structure and organisation of multi-professional and relatives’ involvement in ACP led to fragmentation of the process. This marginalised the voice of both the resident and nursing home staff, thereby limiting ACP as a tool to enhance quality of end-of-life care.

## Key Points

- Structure and organisation of nursing home multi-professional care is disjointed and leads to advance care planning (ACP) being fragmented.
- Power imbalances between nursing home staff and visiting professionals impact sharing of resident wishes.
- Family involvement in ACP is not formally recognised or supported but is important to nursing home residents.
- Fragmentation prevents a resident-centred approach, limiting ACP as a tool to enhance end-of-life care in nursing homes.

## Introduction

Due to an ageing population globally, deaths in older age are rising [1]. Consequently, there is an associated increase in demand for palliative care, which focuses on providing a patient-centred approach to improve quality of life for those nearing the end of their life [2]. In the United Kingdom (UK) residential and nursing homes, as providers of long-term care, are key sources of palliative and end-of-life care provision [3], with one in five deaths in England occurring in these settings in 2021 [4].

A multi-professional approach is a key feature of the internationally recognised model of palliative care delivery [5], with the multi-professional team including health and social care practitioners, and spiritual care providers. Multi-professional care is also recognised as the gold standard for care of older people, being central to Comprehensive Geriatric Assessment [6]. Nursing homes have registered nurses onsite. However, globally, there is variation as to which other professionals are available onsite, and which use the services of visiting professionals [7]. In the UK, other than nursing home nurses, the majority of professionals providing care in nursing homes are from external services, either National Health Service (NHS) or private sector [8].

Providing individuals with opportunities to discuss their wishes for future care is considered an important component of high-quality end-of-life care [9]. Advance Care Planning (ACP) involves discussing and documenting wishes and preferences for future care [10] and engaging with ACP is considered a key priority for nursing homes [11]. ACP conversations address a range of issues that affect all dimensions of human experience and require the expertise of many professional disciplines [12]. Research in both Australia and the UK has highlighted multi-professional involvement as a requirement for successful implementation of ACP in long-term care settings [13, 14].

In the UK, the quality of a nursing home’s interrelationships with professionals from the wider health and social care system determines the quality of the end-of-life care they provide [15]. Evidence suggests that increased end-of-life care knowledge amongst all involved supports multi-professional working, improving communication and working relationships [16–19]. However, stronger frameworks for multi-professional working are also needed to clarify lines of accountability [18, 20], alongside accessible services [16, 17]. There is limited understanding of what multi-professional working relationships or models of care to support nursing homes should look like in relation to end-of-life care and ACP.

This paper reports findings from a doctoral study that explored multi-professional working within the nursing home setting [21]. Using ACP as an exemplar of end-of-life care practice the study investigated multi-professional involvement in ACP within two nursing homes. The study was underpinned by the research questions: (i) What factors influence multi-professional involvement in the ACP process within nursing homes? (ii) How does multi-professional working impact the ACP process in nursing homes? Findings suggest that a disjointed system characterised the process of multi-professional involvement in ACP, which led to fragmentation of ACP. The paper enhances understanding of how the quality of end-of-life care could be improved for nursing home residents.

## Methods

### Study design

An ethnography was conducted to capture a thick description to understand the social processes and interactions that underpin multi-professional working in UK nursing homes. The study involved direct experience and examination of two nursing home settings, focused on understanding the meanings motivating the actions of all involved in ACP [22]. The study was approved by the Social Care Research Ethics Committee (15/IEC08/0004).

### Settings

Two purposively sampled nursing homes were identified via specialist palliative care education facilitators. Characteristics of the homes are provided in Table 1. Before fieldwork commenced, the study was introduced to those living and working in the homes via newsletters and staff and resident meetings.

**Table 1:** Nursing home characteristics

	Nursing Home 1	Nursing Home 2
**Number of beds**	>50 beds Percentage of permanent residents in a nursing bed ranged from 76% to 85%.	>50 beds All residents in a nursing bed.
**Location**	Urban	Rural, on outskirts of a large town.
**Ownership**	Part of a large group of homes (20+)	Part of a small group of homes (<9)
**Length of stay**	Frequent respite or convalescence admissions, with at least 13 during fieldwork.	Two residents had lived in the home for twelve years. Two residents were admitted for respite during fieldwork.
**Staffing**	At least two nurses per day shift, one nurse at night. Some staff turnover observed during fieldwork. Agency staff rarely used; support sometimes provided by other homes in the group.	At least two nurses per shift. Only one leaver and two new starters observed during fieldwork. Several staff had worked at the home for more than 20 years. Agency staff rarely used.
**Multi-professional services**	Residents registered with one of three GP practices in the town. One practice nominated as main practice, but not a contracted service, with between 75 and 85% registered with this practice at any one time. Other professionals known to have visited during fieldwork were specialist nurses (diabetes, palliative care), social worker, occupational therapist, physiotherapist, optician.	Most residents registered with one GP practice contracted to provide medical services, including a weekly GP round. 10–15% at any one time registered with the GP practice geographically closest. Other professionals known to have visited during fieldwork were specialist nurses (Parkinson’s disease, palliative care, community psychiatric nurse, continence), social worker, physiotherapist.

### Participants

All nursing home managers and nurses, and visiting professionals identified as key stakeholders were invited to participate. Other nursing home staff were identified via managers and nurses. Residents approached were identified to ensure different levels of engagement with ACP, visiting professional involvement and prognosis. All visiting professionals involved in the care of a participating resident and relatives nominated by the resident were also invited to participate. Table 2 details inclusion and exclusion criteria. All potential participants were provided with an invitation letter and a participant information sheet.

**Table 2:** Inclusion and exclusion criteria for participants

Participant type	Inclusion criteria	Exclusion criteria
**Visiting health and social care professionals**	Provide care to residents within a participating nursing home. Involved in key ACP activities within the nursing home or involved in the care of a participating resident.	
**Nursing home staff**	Involved in key activities relating to ACP, involved in the care of a resident participating in the study or identified as a key informant by colleagues.	Not directly employed by the nursing home.
**Relatives**	Nominated by a resident participating in the study.	
**Residents**	Resident in a participating nursing home. Had prior involvement in ACP.	Residents who lacked mental capacity to be involved in the ACP process and/or to consent to participate in the study.

Six residents, four relatives, nineteen nursing home staff and seven visiting professionals participated (Table 3). Residents included men (*n* = 3) and women (*n* = 3), aged between 79 and 93. All relative participants were adult children of residents.

**Table 3:** Numbers approached to participate in, recruited to and who withdrew from the study

Participant group	Approached	Recruited	Withdrew
Residents	22	6	0
Relatives	6	4	0
Nursing home managers	4	4	0
Registered nurses—days	15	6	1
Registered nurses—nights	6	0	0
Care assistants—days	14	7	0
Care assistants—nights	1	1	0
Activities staff	2	1	1
GPs	8	3	0
Specialist nurses	4	3	0
Social care professionals	1	1	0

### Data collection

NA undertook fieldwork for between six and seven months in each nursing home. Data was collected using unstructured observation, formal and informal interviews and document review. NA was present in each home for more than 200 hours over more than 50 separate occasions, both day and night and on all days of the week. Decisions regarding when to observe were informed by salient events involving multi-professional working and when participating nursing home staff were on duty. Daily routines of participating nursing home staff; one-to-one or group discussions relating to care provision or care planning between nursing home staff or nursing home staff and visiting professionals; and interactions between nursing home staff and/or visiting professionals with participating residents and relatives were observed. No personal care was observed. Observations were recorded in field notes.

Sixteen individual, audio-recorded interviews of 25- to 40-minute duration, and one with a resident and relative dyad of 55 minutes were completed by NA. The topics used to guide these included views and experiences of ACP discussions, multi-professional involvement in ACP, and barriers and facilitators of multi-professional involvement. Where availability prevented nursing home staff and visiting professionals participating in a formal interview, these topics were explored using informal interviews. These were characterised by asking questions while observing, with data recorded within field notes, and differed from naturally occurring talk when questions asked were led by the situation being observed.

Nursing home documents, such as each home’s ACP and end-of-life care policies, and resident notes were reviewed and re-read at intervals throughout the fieldwork to identify updates. Documents not available in the public domain were included with the written permission of the nursing home manager and resident notes were reviewed with consent of the resident.

### Data analysis

**Figure 1:**
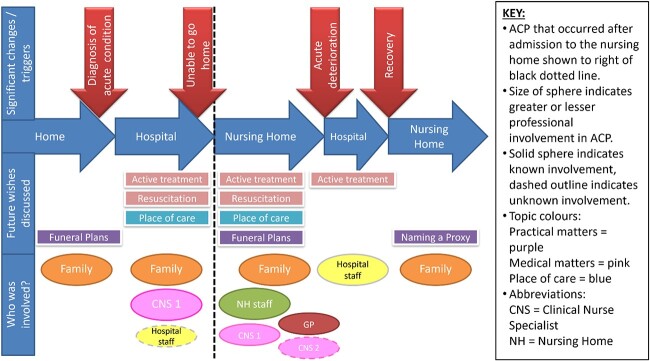
Map of Lily’s ACP trajectory.

An inductive and iterative approach to data analysis was taken integrating:

- Thematic analysis using Braun and Clarke’s approach [23].
- Mapping of resident ACP trajectories by plotting a visual display for each participant resident with the trajectories then compared. Lily’s trajectory is shown in Figure 1.
- Documentary analysis of nursing home internal policies.

This enabled a rich ethnographic picture of the settings to be developed and an understanding of how multi-professional working in relation to ACP was accomplished in each organisation. Combined analysis enabled development of individual resident and organisational perspectives. NA completed initial coding, with theme generation evolved and interpretations tested through discussion with MM. Data was managed using NVivo (version 11) and Microsoft Excel.

Two theoretical frameworks were used as a lens for the interpretation of data. The first ‘Knotworking’ describes collaborative work between loosely connected actors and activity systems [24] involving changing combinations of individuals distributed across time and place [25]. Professionals tie, untie and re-tie otherwise separate threads of activity during their interactions [26]. Second, the practice-based concept of temporal structuring suggests that time is realised through recurrent practices that produce temporal structures [27]. These shape organisational life and are used by individuals to regulate and account for their activities.

### Findings

Three themes were developed through the analyses: Disjointed System, describing the structure and organisation of care; Enacting ACP, revealing how enactment of ACP impacted multi-professional working; and Professional Reach, depicting both the range of people involved in ACP and the scope of their involvement. The three sub-themes of Disjointed System (Relationship continuity, Information sharing and Coordinating multi-professional ACP) and a sub-theme of Professional Reach (Relatives’ involvement in ACP) relate to the focus of the paper and are presented here. Pseudonyms are used throughout.

### Relationship continuity

In both settings, fostering relationships through continuity of professional involvement was valued by residents, relatives and professionals and considered important to facilitate ACP conversations. Relationships with two GPs who provided a weekly ‘ward’ round in one home were spoken about positively by residents, relatives and nursing home staff. Residents and relatives in both settings also acknowledged the need for continuity of nursing home staff:

Jackie [relative] discussed that it was good that the two GPs knew the residents well. She identified that knowing the resident's history was important. She said that ‘continuity is very important’ (Field Notes—Visit 013).

Visiting professionals and nursing home staff talked about ‘getting to know’ residents. Residents suggested that they were more likely to discuss their treatment and care preferences when they ‘knew’ a professional. This was important in the end-of-life care context where the sensitive nature of conversations was underpinned by the wish to hold these with someone with whom they had established a rapport:

‘I mean if you’ve got something wrong with your finger any doctor will help but for other things and the fact that they know you a bit makes all the difference’ (Hilda, resident).

While continuity was important at an individual level it was prone to disruption by the structure and organisation of care between nursing homes and visiting professionals, and the organisation of staff within the homes. Visiting professionals frequently attended on a reactive, *ad hoc* basis. The transient nature of these interactions limited opportunities for trusted relationships to develop.

Regular GP rounds and key worker roles, such as the named nurse approach used in one nursing home, were seen as supporting continuity. Senior nurses or key workers were reported by some visiting professionals as most able to provide informed insight into a resident’s condition. However, the nature of nursing home shift work and reactive professional visits meant that staff members with the most in-depth knowledge of the resident were not necessarily on duty at the time of the visit. For example, the GP round was primarily overseen by senior nurses, but the unpredictable timing of reactive visits between the rounds meant multi-professional collaboration was facilitated by the nurse on duty when the professional visited:

Gemma [nursing home nurse] spoke about being more involved with GPs who visited ad hoc rather than the ‘big round’. She said that May and Gita [managers] are only heavily involved in the round, yet she thought as these [ad hoc] visits were when a resident is poorly, they were more important (Field notes—Visit 042).

Nursing home nurses attempted to influence continuity of GP provision where possible, such as requesting visits from a GP that had seen the resident before. However, this could not always be achieved.

### Information sharing

Successful implementation of ACP within the nursing homes required information to be shared between a number of professionals. Processes such as handovers and written documentation were used to communicate information internally within both settings. However, these were not always effective. For example, handovers usually did not provide information beyond the previous 24-hour period, meaning changes outside that timeframe might not be communicated to all staff. This could have implications for both residents and staff:

‘And you’re not always getting the full handover you see. You’re just picking up bits. When you come in from that handover, you’re just picking up what’s relevant then’ (Sarah, nursing home nurse).

ACP was recorded in residents’ notes, yet it was challenging for staff to remain up to date with changes to residents’ wishes. Temporal structures, such as the expectation that personal care is provided before lunch and medication rounds, meant staff only had time to read resident notes to update themselves if gaps occurred in their usual routines:

Claire [care assistant] told me she was just taking the opportunity to learn more about the residents. She was sat in the office reading resident notes. She was reading the page called ‘All about me’. She said she didn’t often have time to do this, but she liked to know more about the residents (Field notes—Visit 104).

However, Katie, a nursing home nurse, explained that you ‘can’t know everyone’s care plan’ but having wishes documented meant they were available to be referred to when needed.

The regular GP round was the only example observed of a structured multi-professional process for information sharing. Otherwise, sharing of information between nursing home staff and visiting professionals, including that relating to ACP, occurred *ad hoc* such as by telephone. The work required to engage with professionals to share information where services were reactive and difficult to access was a source of frustration for nursing home staff:

‘So much money and resources are wasted just trying to communicate’ (Katie [nursing home nurse], Field notes—Visit 102).

Visiting professionals did not routinely document in nursing home notes unless asked and were not observed recording in the nursing home’s ACP documentation in either home. ACP discussions were recorded in their own notes, leading to silo working and duplication. It was acknowledged that the different organisational ACP documents did not always link together. Although there was a widely held belief that these were shared between organisations, this did not often occur:

‘Saying that, [locality ACP document] doesn’t routinely go to the home. At the bottom of the form there’s the three people you send it to and the home, believe it or not, isn’t one of them’ (Dr Slater, GP).

This could result in there not being one complete record of all wishes and preferences expressed by a resident.

### Coordinating multi-professional ACP

Both ascertaining and implementing a resident’s wishes for future care required coordination across organisational boundaries. A coordinated approach to ACP and end-of-life decision-making was demonstrated by the GP round in one nursing home, offering space and time for communication between nursing home nurses and the doctor. However, planning and decision-making in the two nursing homes generally did not involve more than two professional disciplines. Where more than two disciplines were involved, such as a specialist nurse, GP and nursing home staff, they did not discuss the resident’s care all together or jointly with the resident and relatives. Discussions occurred in professional dyads, often not involving consistent individual professionals.

This complexity is illustrated by the professional involvement in Lily’s ACP, outlined in Box 1. This evolved as different collaborators had conversations to gain understanding of her wishes and preferences. However, professionals worked independently, and this was not always shared with all involved. As a result, Lily’s ACP was fragmented.

Box 1 Professional involvement in Lily’s ACP

- Within the nursing home:
  - Most ACP involved family and nursing home staff.
  - Some involvement of GPs.
  - A community specialist nurse was involved.
- ACP also occurred during a short admission to hospital due to acute illness.
- Some professionals were involved on a temporary basis. For example: the community specialist nurse discussed future treatment options and hospital staff discussed preference for place of care.
- Interaction occurred between the nursing home nurses and GPs during visits, but not between a constant professional dyad. Lily was visited by four different GPs, who each interacted with the nursing home nurse who was on duty when they visited.
- Both nursing home nurses and GPs interacted with the community specialist nurse, but separately. It was not always the same nursing home nurse or GP who interacted with the community specialist nurse.

During observations, it was evident that coordinating ACP was impacted by a power imbalance existing between visiting professionals and nursing home staff. The weekly GP round was afforded special status, use of organisational space for the round was prioritised over other activities and work planned around the unpredictable start time of the round, which depended on when the GP arrived at the home. Visiting professionals visited at times aligned with their temporal structures, such as the typical routine of GP visits between morning and afternoon surgery. Nursing home work adapted to accommodate this, suggesting that visiting professionals’ time was perceived as more important than that of the nurses.

In both nursing homes staff demonstrated deference to visiting professionals, viewing them as experts and needing to justify requesting their input into resident care:

Katie [nurse] said she struggled with Cyril as to whether to call a GP or not. She said he reports symptoms such as diarrhoea or coughing up blood but there is never any evidence. She said he always flushes the toilet and there’s no smell in his room (Field notes—Visit 073).

Visits were not explicitly requested for ACP, not appearing to warrant an external professional’s time specifically for this purpose and was only raised during visits for other reasons.

There was a lack of consensus between professional disciplines regarding who was responsible for coordinating ACP. During interviews a GP and a specialist nurse each described it as their role to lead on this, even though their visits to residents were sporadic. This reflected a perception that nursing homes and their staff had a low status within the health and social care system, which nursing home staff referred to:

Discussing a challenging professional visit, Phoebe [manager] said that generally the NHS treat nursing home staff as lay people and therefore that they know nothing (Field notes—Visit 066).

In practice, ACP was primarily undertaken by nursing home staff unless involvement of other professionals was actively sought.

Residents had the least power to influence professional involvement in their ACP. Their access to visiting professionals was organised by nursing home staff through GPs who made most referrals. Residents were not always directly involved in these consultations and only became aware that issues they had raised were addressed when they were resolved:

Hilda said she had requested an increase in the dose of her antidepressant a short while back, but this had all been sorted out by the nurses faxing her GP. She reported this as ‘not satisfactory’ as her GP hadn’t been to see her. She didn’t think a nurse could really explain how someone else is feeling (Field notes—Visit 102).

### Relatives’ involvement in ACP

Involvement of relatives was a consistent feature of the ACP trajectories for all six resident participants. Prior to admission to the nursing home ACP had often been discussed solely with relatives and once living in the nursing home, some residents valued ACP discussions with their family more than talking to professionals:

‘I think just the role that I’m in now, that as soon as somebody asks him a question, he talks to me about it’ (Monica, daughter).

The ACP topics residents discussed with relatives included clinical decisions such as resuscitation:

May [manager] told me that the request for the DNACPR [Do not attempt cardiopulmonary resuscitation] form completed today had come from the daughter. The daughter had discussed it with the resident (Field notes—Visit 048).

Nursing home staff often included relatives in ACP discussions with residents, whereas visiting professionals usually only engaged relatives if they happened to be present at the time. The GP round enabled involvement of relatives as its time was more predictable than *ad hoc* visits. Involvement of relatives in ACP discussions was perceived by nursing home nurses and GPs as mitigating against risk of conflict when a resident was unable to make decisions or confirm previously expressed views:

‘But I think the real big advantage is that the resident and their family are all in agreement because one of the worst things is when you’ve got family saying no, no he has to go to hospital, … and you know that’s not what the resident wants’ (Laura, manager).

Being present during visits or having direct communication with visiting professionals was sought by many relatives. Relatives were observed to influence multi-professional involvement in ACP, asking nurses to arrange visits or making direct contact with a professional:

‘Some families would like to have the GP because they would like to get the, his input on that’ (May, manager).

Relatives also influenced whether a resident’s expressed wishes were honoured. Staff in both homes described what they perceived to be relatives’ unrealistic expectations about health outcomes, and which did not align with those of professionals. This could lead to residents being admitted to hospital contrary to previously expressed wishes and not achieving a ‘good death’:

There was a discussion between May, Sarah and Christine [nursing home staff] about relatives and them not wanting residents to die. They discussed unrealistic expectations and how that could make it difficult for them to achieve what they considered a good death for residents (Field notes—Visit 044).

Although nurses and GPs considered some relatives to have expectations that were not viable or idealistic, actively managing these concerns was not prioritised. This was despite both nursing home staff and visiting professionals recognising that relatives may need support coming to terms with resident ACP decisions:

‘Do we support families as much as we should and could? Maybe not. … You know you concentrate on the patient or the resident and actually … it’s got to be a whole family, whole family approach’ (Laura, manager).

## Discussion

The focus of this paper has been on how nursing home staff, visiting professionals and relatives worked together to support ACP in two UK nursing homes. The use of ethnography enabled rich and detailed insights to be gained within and across different settings. This helped to identify and characterise the dynamic social processes and interactions within each organisation that influenced multi-professional working in ACP. Findings demonstrate multi-professional and relatives’ involvement in ACP was disjointed, with professional processes both internal and external to the nursing home not well aligned. Researcher influence on the findings is acknowledged. NA is an experienced specialist palliative care nurse and at the time of the study one of her parents was being cared for in a nursing home. These subjectivities will have informed interpretation both in the field and during analysis, and NA kept a reflexive diary to support the identification of tacit knowledge from her own experience informing interpretation. Sharing of information about NA’s clinical role in the participant information sheet may also have influenced participants’ behaviour.

Using the lens of temporal structuring [27] has shown how time for ACP was understood and limited by embedded temporal structures that regulated activities of both nursing home staff and visiting professionals. The temporal structures of the nursing homes and those of visiting professionals did not interrelate easily, such as the mealtime schedule and the well-established temporal structure of a typical GP’s day. This misalignment impacted both multi-professional working and the perception of time availability to become involved in ACP. The only temporal structures that incorporated ACP to any extent were the GP round and the care planning process in each home. Previous research has highlighted a need for sufficient time to facilitate ACP discussions [28], but this has not been explored beyond the potential constraint on ACP in practice. Internal nursing home temporal structures also hindered enaction of strategies to bridge gaps in continuity. Staff reported that being ‘time poor’ prevented them from updating themselves, except through handovers. Enactment of alternative temporal structures may be possible; however, highly institutionalised and widely recognised social practices, including organisational routines, are hard to change [27].

Multi-professional input to residents’ ACP could involve many different professionals across different settings, as for Lily (Figure 1 and Box 1), which can be described as a knotworking approach to multi-professional working. This approach impacted continuity [24], considered important for ACP. It affected both continuity of relationships between professionals and residents, and availability of information so there was continuity of care management that aligned with resident preferences. Implementing ACP in practice has been acknowledged as challenging because the relational work and continuity of care required for these complex and unpredictable conversations is often not prioritised [29]. However, findings from this study suggest that how multi-professional support to the two nursing homes was structured and coordinated was also not conducive to this. The knotworking approach to multi-professional working meant that visiting professionals and nursing home staff rarely worked collectively on ACP. ACP information could be duplicated, being discussed seperately with more than one professional, and could also be recorded in silos, with information not being shared and residents’ preferences not adhered to.

This highlights the need for effective information sharing or a shared ACP document to align involvement of different professionals. Such communication tools or documents are considered crucial to the success of knot-based teamwork, being important to coordinate actions [26]. Within the nursing homes, whilst senior nurses or keyworkers were likely to be well informed about individual residents, shift patterns meant they were not always on duty to inform decision-making with professionals, leading to a reliance on information contained within documents. However, both the nursing homes and the local NHS systems had different documents, and these were not routinely shared. Organisations took ownership of ACP based on completion of their documents, and, in this way, control over the ACP process was exercised through their documents. There was power imbalance with NHS staff having access to a GP initiated locality document, but care homes not included on the circulation list for this document, challenging achievement of effective information sharing. This confirmed the low status of nursing homes in the professional hierarchy and supports previous research that has shown them to be marginalised from whole systems approaches and that health sector staff often have low levels of respect for the experience, knowledge and skills of nursing home staff [30, 31]. Power imbalance has also been shown as a barrier to effective inclusion of nursing homes in the whole-system approach to sharing of ACP information via Electronic Palliative Care Coordination Systems [32, 33].

This hierarchy was less apparent in the weekly GP round, where a more equal platform and joint decision-making was demonstrated, relationships between the nursing home staff and GPs having developed over time. However, the GP round was an exception with an otherwise absence of a forum for professionals to have these discussions. The GP round was also limited to nursing home nurses and GPs. Research provides some evidence to support the use of a forum for multiple disciplines along with residents and relatives to discuss ACP [34–36]. Implementation of the Enhanced Health in Care Homes (EHCH) Framework across England since 2020 (after data collection was completed) includes a weekly multidisciplinary team (MDT) round [37]. However, the EHCH Framework does not specify inclusion of residents and/or relatives in these MDT discussions, unlike case conferences [36, 38].

A case conference approach would better align with the finding that relatives have an important role in ACP for nursing home residents and formalise their involvement. Involving relatives in proactive planning has been shown to lead to shared understanding and acceptance of the medical situation [39] and greater involvement of relatives in decision-making [38], with the potential to reduce conflict with families [40]. The study findings also illustrate how power imbalance can mute the voice of the resident. A case conference model for ACP would enable delivery of a more resident-centred approach, given ACP is identified as important for delivering person-centred care [41, 42].

Findings revealed a lack of clarity as to who had overall responsibility for coordinating ACP in both nursing homes, which threatens to undermine ACP. This reflects previous research suggesting no one professional group has responsibility to initiate, lead or coordinate ACP [43]. As ACP is an iterative process [44], with findings highlighting that this happens both over time, in different settings and involving professionals from many organisations alongside relatives, its coordination is complex. Although GPs are frequently identified as being well placed to lead ACP [33], they rely on the knowledge of nursing home staff to inform their decision-making [45]. This suggests nursing home staff may be better placed as coordinators. They consider acting as advocates for their residents to be part of their role in providing end-of-life care [46] and leading and coordinating ACP would better enable them to do this. However, achieving this relies on developing and sustaining relational working between visiting health professionals and nursing home staff, identified by both this study and previous research as important [47].

### Strengths and limitations

Using ethnography enabled in-depth investigation, providing a cultural perspective on multi-professional working in ACP in the two nursing homes. Multiple methods of data collection and including multiple stakeholder groups provided depth and breadth of insight.

The study was limited by recruitment only of residents who had mental capacity to both complete ACP and consent to the study. No participant residents had a diagnosis of dementia, yet a substantial majority of nursing home residents in the UK have some cognitive impairment [48]. Data regarding residents with dementia was therefore only obtained indirectly as part of discussions with professionals. This limits the transferability of the findings to other settings, specifically those nursing homes working solely with residents with cognitive impairment.

### Implications for practice, policy and research

The study findings suggest that inter-organisational strategies to achieve more integrated ACP processes between nursing home staff and visiting professionals across local systems, with nursing homes engaged as equal partners in development of these, would reduce fragmentation. Offering opportunities for joint conversations between visiting professionals, residents and relatives, involving relatives more formally in ACP and providing support where required for goals of care to be agreed could also enhance ACP. Further research is required into the benefits of case conferencing in UK nursing homes as an approach for this.

Current policy promotes ACP as giving control to the individual, but the findings show that ACP for residents in the two nursing homes was often controlled by organisations and by professionals. ACP policies should put the resident at the centre, with more research required to understand resident experiences of locality-wide ACP initiatives. However, the findings also suggest that policy which ensures care within nursing homes is provided by professionals who know residents well may be more beneficial than policy to ensure implementation of one approach to ACP.

## Conclusion

This study has shown ACP taking place in a disjointed system of multi-professional working in two UK nursing homes. The structure and organisation of both nursing homes and visiting professional care disrupted continuity and limited information sharing and coordination of ACP. Residents wished for their relatives to be included in ACP discussions with professionals but formalised approaches to achieving this were limited. Multi-professional working and enactment of ACP was influenced by an imbalance of power that led to ACP and end-of-life decision-making being fragmented with marginalisation of the resident and nursing home nurse voice. This limits ACP as a tool to enhance quality of end-of-life care.
